# Hospital and Institutional News

**Published:** 1912-08-10

**Authors:** 


### HOSPITAL AND INSTITUTIONAL NEWS.

### SUSPECTED CASES AND LIVERPOOL HOSPITALS.

A boy, aged seven, who was admitted to the Royal Infirmary, Liverpool, on July 25, and operated upon for supposed appendicitis, had also a gland, the presence of which was not previously known, removed from the groin, and this was found to contain bacilli that, from laboratory investigation, seemed to indicate bubonic plague. When this was discovered he was immediately isolated, and three days later was removed to an infectious diseases hospital. The source of infection is supposed to be the bite of a flea carried by a foreign rat, which gains probability from the fact that the boy's home is near the docks. The unwitting admission of a case of plague into a general hospital has led to a strict watch by the medical staffs at the Liverpool hospitals in the case of certain patients. A suspected case of plague was discovered at another hospital a few weeks ago. No further case happily has occurred, and the possibility of the spread of the disease has been very carefully guarded. Hospitals in the great port cities are unpleasantly reminded of the added responsibilities thrown upon their medical staffs by suspected cases of this sort from time to time.

### SET DIETS FOR MENTAL WORKERS.

The improvements in the conditions of life affecting those who are on the staffs of our mental hospitals are always worth attention, even if they seem small in themselves, for the strain on mental workers is probably more acute than on those in any other type of institution. In Dr. J. Francis Dixon's recent report on the West Humberstone Mental Hospital, Leicester, the first which he has issued since his appointment in November last as medical superintendent in place of Dr. Finch, who then retired after forty-two years' service, he refers to certain changes in diet which have been effected. The object of these was to improve the quality and also the variety. This, says Dr. Dixon, has been gained, but he suggests a further improvement. Why not, he urges in effect, adopt "a number of set diets which would meet practically all cases and result in the abolition of extras?" It would be very interesting to learn whether this experiment is now being carried out, and Dr. Dixon would earn the thanks of many a superintendent if he would make known the set diets which have been decided upon at West Humberstone. Variety in diet is still too often an unsolved problem in many institutions, and the interchange of successful experiments is the true meaning of institutional solidarity.

### A WAR OFFICE CLAIM ON AN ISOLATION HOSPITAL.

The Isle of Wight Joint Isolation Hospital Board, at a recent meeting of which Mr. J. I. Barton presided, had to consider a curious proposal from the military authorities. It was contained in a letter to the effect that if no charge were made for the servants of the well-to-do ratepayers in the isolation hospital, the War Department claimed the right to free treatment for the soldier and his family. After reading this request it was decided, on Mr. Barton's suggestion, to send a letter to the Secretary of State for War pointing out that the case of the soldier was not analogous to that of the ratepayer's servant, since the Government only paid a portion of the rates in respect of military establishments. The reply was also to add that it would be very unfair for a small hospital to have to receive a large number of soldier patients free of charge. The War Office has the reputation of cheeseparing economy in many matters. The Hospital, in fact, drew attention to a very glaring instance at Parkhurst Military Hospital in the Isle of Wight some time ago, and this suggestion is not likely to detract from it.

### THE NEW PRESIDENT OF OLDHAM INFIRMARY.

The new president of Oldham Infirmary, who has had suddenly to be appointed to fill the vacancy caused by the death of Mr. James Dodd, is Mr. J. W. Taylor, J.P. He has long been connected with the institution, and has practical experience of its management from having acted as vice-president on the house committee and as chairman of the board of management for many years. It was, in fact, in 1885 that Mr. Taylor first joined the board, in the days when Mr. W. Richardson was president. Since this date three presidents have come and gone. In an interesting speech acknowledging his election Mr. Taylor said: "The infirmary is not sufficiently well-known by the townspeople. The more they come and see the work being done the more highly the people will appreciate it. They had made great strides during the last twenty-seven years, but there was one thing he would like to see done in connection with the infirmary. Those who visited it and saw the patients from the time of convalescence, when they were beginning to feel themselves better, would see they required something more when they had to go home. He was speaking more particularly of mothers. If some kind-hearted townspeople would only take up the question of providing somewhere in their midst a convalescent home, where those people whom he had spoken of could go when they had been sent out of the infirmary, it would be a splendid thing. He hoped before many years had gone over they would see something of the sort." Mr. Taylor then moved that Mr. H. L. Hargraves, J.P., be appointed vice-president.

### A DOCTOR-DIPLOMATIST IN CHINA.

The appointment of Dr. G. E. Morrison as political adviser to the President of the Chinese Republic is a striking example of the part which members of the medical and hospital professions are increasingly beginning to assume in public life. A colonial by birth, for he was born in Victoria in 1862, Dr. Morrison studied at Melbourne and Edinburgh Universities, and graduated M.B. and C.M. at the latter in 1887. The instinct for travel had already shown itself, for he was a pioneer in

New Guinea at the age of twenty, during which expedition he was wounded, the spear-head being eventually removed by Professor Cheyne at Edinburgh. As a peripatetic physician, now in practice and now on his travels, by way of Paris, Spain, Morocco, and the United States, Dr. Morrison arrived in the Far East. Here his walking exploits attracted notice. From Normanton in the north to his home in Victoria he walked in 1882, doing the distance of 2,043 miles in 123 days. In 1894 he walked from Shanghai to Rangoon. China seemed to attract him inevitably, and in 1900 his services during the siege of the Legations in Pekin brought him further into notice. Since 1897 he has served as correspondent of the *Times* in Peking. The appointment has been very popular among the Chinese, who realise that Dr. Morrison's knowledge of Chinese affairs surpasses that of other foreigners. With Dr. Morrison's example to the profession abroad, and Sir Thomas Crosby's at home, medical men have placed before them two notable instances of the position in public affairs which they have so often deemed beyond the purview of medicine.

### THE ROYAL MEDICAL BENEVOLENT FUND.

The British Medical Benevolent Fund has now been granted the title of Royal, and will therefore be known as the Royal Medical Benevolent Fund. Sir John Tweedy is the president, and the work of the Fund has grown quietly since 1836, when it was founded for the relief of distressed qualified members of the medical profession. The widows and children of these may also benefit from the Fund's grants, and annuities are granted to suitable applicants if sixty years of age. Two years ago 197 grants were made and 127 annuities were distributed, the latter ranging from £10 to £36. Every well-ordered profession should have, and happily many do, a benevolent fund of this kind, which ought particularly to appeal to the sympathies of those exceptionally successful members, who should realise more poignantly than most how severe is the struggle and how heart-breaking often the luck which influences success or failure when measured by income. The new title is more dignified, as well as less cumbrous, than the old, and we hope it will mark the opening of a period of extended and still better organised help.

### GOVERNMENT AID FOR COUNTY COUNCIL SANATORIA.

Since last week the intentions of the Government with regard to the assistance that they propose to give to county councils towards the annual cost of maintaining the sanatoria or institutions to be erected under the Insurance Act have become known. Mr. Henry Hobhouse has received from Mr. Lloyd George a letter, in the course of which he says: "I gathered [from the speech of Mr. Hobhouse we reported last week] that local authorities are prepared to bear 25 per cent. of the annual cost of schemes, if the remainder were provided from other sources; and that their request is that this should be paid to them direct by the Local Government Board. The Government are prepared to go a long way towards meeting your requests. . . While the [Insurance] Bill was passing through Parliament provisions were inserted for extending sanatorium benefit to dependants of insured, and in view of this the Government consented to bear one-half of any deficit in regard to sanatorium benefit where local authorities undertook the other half. It is now urged that schemes for the treatment of tuberculosis should relate to the whole community, and that generally they should be organised and carried out by the councils of counties and county boroughs. This extension involves additional outlay, and in view of this the Government have decided to place at the disposal of the Local Government Boards of the three kingdoms annually a sum of money which will represent approximately half the total estimated cost of treating the non-insured persons as well as the dependants of insured persons. . . As regards the cost of treating insured persons, the sum already provided under the Insurance Act, which, as I have already stated, is about one million pounds, can only pass to local authorities in pursuance of agreements made between them and insurance committees."

### SANATORUM BENEFIT IN PRACTICE.

The administration of sanatorium benefit, on which so many questions are being asked, is established by a circular letter issued to Insurance Committees which gives instructions as follows:—A person applying for sanatorium benefit must obtain a form (Form Med. 1) from the clerk of the Insurance Committee of his district, and furnish evidence that he is insured and suffering from tuberculosis, as certified by his own medical attendant or by the tuberculosis officer. The applicant next takes the form back to the clerk of the insurance committee, who instructs him to obtain a detailed report from a medical practitioner on Form Med. 2. This is a lengthy form relating to symptoms and physical signs. A blank is left as to the fee for filling up this report, but five shillings is suggested as a suitable amount. The report is to be sent to the medical officer of health if a tuberculosis officer is not available. In the case of the tuberculosis officer (or the medical officer of health) certifying the existence of tuberculosis in the applicant, he adds the kind and duration of treatment immediately required (viz., sanatorium, hospital, dispensary, or domiciliary). The fullest details are required respecting home treatment by an order dated July 26, 1912. The medical practitioner is to attend as often as is required and to give instruction to the patient; to keep a scheduled card on one side of which is a detailed account of the condition of the patient and the family and personal history; on the other side the clinical history. The medical attendant is to show this card to the consulting officer, to whom a report must be sent not less than once in three months. The administration of sanatorium benefit as above outlined seems to be on a satisfactory basis, and the general confusion in the public mind justifies this account of its method.

### SUGGESTED DEVELOPMENT OF THE BATH TREATMENT.

Sir William Ramsay is understood to have accepted for five years an engagement as scientific adviser to the Radium Development Syndicate, which is negotiating for a lease of the baths at Bath. Sir William Ramsay has made important investigations into the hot mineral waters of Bath, and his advice will be at the disposal of the Syndicate, if its offer be accepted to spend not less than £250,000 on the bathing establishment.

### RUBBER COMPANIES AND TROPICAL MEDICINE.

The appeal for establishing the London School of Tropical Medicine upon a sound basis is being prosecuted steadily. Mr. Austen Chamberlain's aid has been gained, and at a meeting at the London Chamber of Commerce, over which he presided, it was decided to form a City Sub-Committee to co-operate with the General Committee in organising a systematic appeal in support of the fund. "Several companies, particularly rubber companies," Mr. Chamberlain remarked, "which had been approached by one of their members, Mr. Bride, had generously responded to his suggestion. Additional subscriptions had been received from some of those who had already contributed, and contributions had been promised by others who had not previously sent donations. The appeal was opened in the early part of this year under the auspices of the Lord Mayor by a meeting at the Mansion House, and a sum of £4,000 was contributed. The prosecution of the appeal had to be suspended owing to other claims on the City of London, but it was resumed about a month ago, and the total amount received to date exceeded £28,000. Of that sum something over £19,000 had been contributed in response to personal letters which he had written in the course of the last month or five weeks. He only mentioned that because he was convinced that an appeal of that kind could be prosecuted successfully on one condition only, and that was that it was made a personal appeal." Mr. Chamberlain is perfectly right. The success of such an appeal depends upon its personal nature and upon the understanding of those who, like the rubber companies, realise the personal importance to them of its objects.

### MENTAL HOSPITAL DOCTOR ARRESTED AS A "SPY."

Among the party of five English yachtsmen who were arrested near Kiel on a charge of espionage last Saturday was Dr. N. Roberts. Dr. Roberts is a member of the staff of the Horton Mental Hospital, to which he was recently appointed by the Mental Hospitals Committee of the London County Council. Previously Dr. Roberts had been on the staff of the Cane Hill Mental Hospital for eleven years. Mr. James Moody, the medical superintendent at Cane Hill, describes the whole charge as preposterous, and says that, while the party may have been a little indiscreet in taking photographs, to call them spies is ludicrous.

### THE OTHER SUSPECTS AT KIEL.

Another member of the suspected party is Dr. D. M. Stone, now house surgeon at the Metropolitan Hospital, who was educated at St. Bartholomew's, which institution is indirectly concerned in "the Kiel spy case" from the fact that Dr. Alan Hilary Moore, house physician to the hospital, was to have formed one of the party for a fortnight had not his hospital duties entailed his return and saved him from what is proving not so much a peaceful holiday as a desperate enterprise. Dr. Stone has the added misfortune of being an amateur photographer, and had informed a friend by post that he had taken "about three dozen photographs," which he intended to use for a newspaper competition. In view of his arrest, however, the intended publication of his photographs in a daily paper would seem to have been fraught with international complications of a grave kind! The party of yachtsmen intended to pass the Kiel Canal *en route* to the Baltic, and, if the weather were favourable, to cruise in *The Silver Crescent*, a yacht of twenty-seven tons. On learning of the arrest of his friends Dr. Moore at once went to the German Embassy. He has since described the part of the trip at which he was present, and detailed the subjects of the photographs for which he was responsible. Dr. Stone, who previously held the post of assistant house surgeon, in the ordinary way was expected back at the Metropolitan Hospital on Monday next. His absence, anyway a serious inconvenience, will prove doubly so at this time of the year, when everyone is taking his holiday, and vacancies on the hospital staff are therefore doubly hard to fill.

### THE LEEDS INFIRMARY EXTENSION SCHEME APPROVED.

After a protracted discussion the Leeds Infirmary Extension Scheme, which involves, as already pointed out in these columns, the co-operation of the Corporation, has been approved by the City Council. A special committee has been considering the extension and the construction of the new street which it would necessitate, and the Lord Mayor, Mr. William Nicholson, moved to confirm the proceedings of the committee. "The infirmary board," said the Lord Mayor, "has done a great deal for Leeds, and is proposing to do a great deal more, and I think we ought to assist them with all our power in the noble work they are doing." In conclusion, the Lord Mayor said: "I would like to pay a high compliment to Mr. Charles Lupton," the treasurer of the infirmary, "for his skilful purchases and for the very hard work he has done in this matter. I think it is worthy of the best traditions of our public life." After Mr. George Ratcliffe's amendment for postponing the matter had been withdrawn, the resolution was eventually carried by 42 to 11 votes. It is not a little interesting to note that the two most important hospital extensions of the year—that now completed at Bristol Royal Infirmary and that now approved at Leeds—have involved in both cases the destruction of a large area of slum property and a great improvement to the health and amenities of the two cities.

### A FOUNDER OF THE SCHOOL OF MEDICINE FOR WOMEN.

One of the founders of the School of Medicine for Women has died in the person of Mr. Arthur Trehern Norton, C.B., F.R.C.S., in his seventy-second year. He began his studies at St. Mary's Hospital, to which he became consulting surgeon, and was examiner in surgery for Durham University. The development of the Volunteer Medical Service was an object in which he was greatly interested, and he became Surgeon Lieut.-Colonel of the Corps, receiving the C.B. in 1897 in recognition of his services in its formation. For services in regard to the ambulance arrangements during the Franco-Prussian War he was decorated by the French War Office.

### MR. NORTON'S WORK AS DEAN.

In his connection with the founding of the London School of Medicine for Women Mr. A. T. Norton conceived the idea that if a full course of study were provided and the lecturers already recognised as teachers at Metropolitan schools, one or other of the examining boards would accept their certificates from women as well as men students. A provisional council was formed in 1874 to carry out this idea; Miss Jex-Blake secured the association at this council of the leading physicians and surgeons of the day, among them being Mr. Norton. With two others he became treasurer of the moneys entrusted to found the School of Medicine for Women, and at the same time he took up the appointment of lecturer on anatomy, and, on the illness of Dr. Anstie in the same year, came forward to fill the post of dean of the school. Owing to pressure of other duties Mr. Norton resigned the post of dean in 1883, having held it for nine years, covering the most critical period of its existence, during which time he rendered very valuable help to the struggling institution. He was succeeded by Mrs. Garrett Anderson, M.D. Mr. Norton was a member of the executive council until 1890, and on resigning became one of the governing body as a member of association.

### THE LESSON OF THE FIFTH OLYMPIAD.

Considerable and general attention has been aroused by the failure of English athletes to outshine their rival competitors at the Olympic Games held this year in Stockholm. We are treated to jeremiads on national deterioration; as if the unsuccess of certain teams arbitrarily chosen by certain societies and sporting associations can honestly be regarded as evidence of real degeneration of national stamina. Originally the Olympic Games were entirely national. They were a test of the endurance and fitness of trained experts belonging to the same race, educated under more or less the same conditions, and handicapped by the same racial and environmental difficulties. To-day the matter is far different. The Games are no longer national; they are international, and the competitors are recruited from all ranks and trained under widely different conditions. Yet we may grasp one or two lessons from the Fifth Olympiad. One of these is that if a nation systematically trains her manhood in exercises which have always been considered manly she stands more chance to evolve exceptionally competent athletes than if she left such training to individual effort. The provision of public gymnasia, adjunctory to public swimming baths, will do more to ensure such training than the expenditure of thousands of pounds on the shaping of certain selected teams. Such gymnasia should be free and open to every man or youth who cares to enter them, and instruction should be given throughout the day and in the early part of the evening by a competent paid instructor. Abroad, in Germany and Austria, where such gymnasia already exist, public enterprise has been stimulated by the privately organised "Turnvereine," which have done so incalculably much to improve the physical condition of the people. In England we have nothing that in the least corresponds to these associations, with their democratic management and their huge membership. We waste our effort on expensive sporting hobbies, such as football and cricket, whose value is manifestly inferior, from a national point of view, to that of organised and systematic bodily training of at least 50 per cent. of the male community. The Fifth Olympiad has at least called attention to the desirability of devoting more time and consideration to the problem of training the male community in physical exercises. The establishment of a national gymnastic association on the model of the "Turnverein" is a suggestion that we submit to those who are interested in the question. Its adoption would be cheaply purchased by the individual failures which marred our efforts in contending for prizes.

### SECRET REMEDIES: FOR THE DEFENCE.

The Select Committee on Secret Remedies has adjourned until after the Parliamentary recess. It is expected that the deliberations of the Committee will continue until the end of the year at least, as many more witnesses have to be heard. In addition to further evidence from medical practitioners and other witnesses opposed to quackery, evidence will be given by proprietors of secret remedies and others interested in the traffic.

### SUDDEN DEATH OF A HOSPITAL DISPENSER.

On Sunday morning, July 28, a message was received at the West End Hospital for Diseases of the Nervous System that the dispenser, Mr. Arthur Brunyee, had died suddenly. After doing some gardening on Saturday evening, Mr. Brunyee took out his cycle, but feeling unwell he dismounted, and asked for support from a constable. Speedily losing consciousness, he was taken to his home in Harrow, and died early on Sunday without recovering consciousness. Mr. Brunyee's appointment as dispenser dated from January 1, 1899; he also acting as masseur. In May 1903 the appointment of electrician was added. He had thus served the hospital for some fourteen years, and was the oldest servant of the institution still on the active list. He was forty-seven years of age, and leaves a widow and four children. The funeral took place at Pinner Road Cemetery on Wednesday, July 31, at 12.30 p.m. Notwithstanding the early hour and the extremely inclement weather there was a large attendance. Many tributes of regard had been received at the hospital from members of its committee and staff and from wholesale chemical firms.

### WHO'S WHO IN THE PROFESSION.

A new medical reference book, entitled the *Medical Who's Who* has now appeared, published by the London and Counties Press Association, Ltd., of 6 Henrietta Street, Covent Garden. It is not, as its title implies, a selection of the most famous names of the medical profession, but aims at being comprehensive, though its first issue has certainly not the fullness to be expected of later issues. It is in substance a short medical directory, with the personal details that form the basis of the work from which it takes its title. This is the age of reference books, and conservative people who profess regret for their numbers on the ground that personal advertisement is on the increase, would do well to remember that in days when all of us can be approached by telephone, to the annihilation of a not generally regretted former privacy, our personal records also must yield to the claim of social convenience. Our chief criticism of this work therefore concerns its price, 10s. 6d., which may well preclude the popularity at which it aims.

### DOCTORS' FEES IN COTTAGE HOSPITALS.

Considerable friction was manifested at the recent annual meeting of the Lyme Regis Cottage Hospital, Dorset, an illustrated article concerning which appeared in The Hospital last December. The payment of doctors' fees by patients who can afford to do so was the subject of a heated discussion, the Mayor, Mr. A. J. Woodroffe, who is also chairman, supporting an alteration in the rules by saying: "There is a very strong feeling that the hospital should be run for what it is intended, a charitable institution, and not a private nursing home." He proposed, therefore, as the committee unanimously recommended, to amend Rule 13, which at present read as follows: "In-patients belonging to the district will be required to pay a weekly sum of not less than 3s. The actual amount dependent on their circumstances. . . . Such in-patients as can afford to do so shall pay their doctors' fees, but shall be clearly informed as to this at the time of admission. . . . But in special cases, if there is room in the hospital, private patients may be admitted on condition they pay their own doctors' fees and a weekly sum to the hospital of not less than 15s." He now proposed to amend this rule by substituting the following words in the third sentence: "Such in-patients only as can afford to go into the private ward shall pay their doctors' fees." Dr. Barratt Hine said the statement that the Cottage Hospital was run as a private nursing home was ridiculous. He maintained that the accusation had been flung at him that he charged nearly all his patients. Of his nineteen patients sixteen were not charged. Dr. Cooper said he did not exactly see why the medical staff should be dictated to as to whether they should charge fees or not. He contended that they were badly treated by the committee generally. The hon. consulting surgeon got all the thanks and the rest all the blackguarding. Finally, the revision of the rule was carried, with the addition of words allowing the doctors to be able to charge anyone who paid 10s. a week as an in-patient. On the committee being elected the whole of the existing ladies, except one who was not present, declined to serve again. This hasty decision has probably been already regretted.

### THE "JUST DISPOSAL" OF A LARGE ESTATE.

The late Lady Northwick, who has left estate approximately worth £200,000, did not forget cottage hospitals in her will. She bequeathed £500 to Moreton-in-the-Marsh Cottage Hospital, £500 to the Burford Cottage Hospital, near Tenbury, £300 to the Worcester General Infirmary, and £300 to the Oxford Eye Hospital. If these sums seem small in the light of the statement at the beginning of her will, that "with regards to this world's goods I am deeply sensible that I have ten talents to account for. I earnestly hope . . . I am now about to make a just disposal of what I have to leave to others," it must be remembered that the value of a bequest depends as much on the size of the institution as on the size of the gift. Nevertheless, it is probably true to say that the principal part of bequests to charities come not so much from the aristocracy as from the successful adventurers of commerce, titled or untitled, and from the prosperous members of the middle class.

### THE REGULATION OF INFLAMMABLE CLOTHING.

It is an all too familiar story to coroners' juries that fatal accidents frequently occur through the inflammable nature of the fabrics of which the poorer classes make clothing for their children. Chief amongst the materials that account for this loss of life is "flannelette," a material made from cotton which has nothing in common with flannel except that it can be made into garments. Within a period of nine months ending last March, "flannelette" accounted for 133 deaths in this manner, out of a total of 384 reported through burning accidents in the United Kingdom. Juries, medical officers of health, coroners, and philanthropists of many kinds have united to press upon the Home Office legislation designed to reduce this terrible mortality. Indeed, a Departmental Committee of that Office has sat and has suggested certain regulations, but nothing has been done. But we note with satisfaction that a petition signed by various men and women of public reputation—most of whom are medical practitioners—has been presented to the Home Secretary. The wording of the appeal is studiously moderate. The safeguards suggested in the memorial are borrowed from the the Report of the Home Office Committee, and probably represent as much as there is any chance of obtaining at present. The principal regulation urged is one which shall either prohibit altogether the use of the misleading term "flannelette," or shall require the addition of words indicating that the material is made of cotton only. The former would no doubt be an excellent rule, but the latter would, we fear, not convey any very effective warning to poor mothers.

### DINNER TO A HOSPITAL SECRETARY.

The public notice which was taken at the time of Mr. R. A. Owthwaite's recent retirement from the secretaryship of the City of London Lying-in Hospital has been followed up by a dinner and personal presentation, which took place in his honour last week at the Latin-British Exhibition. On the invitation of Mr. J. Francis, the hospital's chairman, the committee and trustees assembled, and after dinner on their behalf Mr. Francis presented Mr. Owthwaite with a silver rose-bowl, which he acknowledged in a short speech.

### THE CO-ORDINATION OF DAY NURSERIES.

In our article on "The Management of Crèches and Day Nurseries," which appeared in April last, we drew attention to the fact that at present no apparent means exist for closing crèches that are badly managed. The only alternative at present is to have some co-ordinated system of inspection, which is not an easy matter in a country like England, where day nurseries are invariably the product of private enterprise or generosity. It is in some degree as fulfilling this need that the work of the National Society of Day Nurseries deserves attention. The Society's inspection applies to all crèches which are affiliated to it, and these at present number 51. A standard of affiliation is secured by a series of questions which have to be answered by a responsible person on the committee of the crèche and by the district medical officer. The Society's Inspector, Mrs. Meyer, makes her report, and if this be approved the crèche receives a certificate and a grant of two guineas. The standard is maintained by the certificate having to be renewed every year. The Society's publication, "Hints on How to Start a Crèche" and the quarterly magazine, *The Crèche*, contain practical information; and, considering the valuable work that remained undone until the Society attempted to bring order out of the chaos that prevailed, it is astonishing that the Secretary, Mr. Herbert H. Jennings, has to complain of a shortage of income. Still a centre of information and organisation has been established, which in itself is a great gain.

### A SUBSTITUTE FOR STREET COLLECTIONS.

The increasing difficulty of securing lady volunteers to conduct the Saturday street collections, and the fact that this method of collecting funds has become somewhat unpopular, have led the Committee of Walsall and District Hospital to abandon these collections this year, and to substitute a house-to-house "envelope" collection. For the purposes of the new scheme the borough was divided into districts corresponding to the municipal polling areas, each of which was placed under the charge of a lady president, who undertook to allocate sections to her staff of helpers. In this way a personal canvass of every household in the borough was secured. Altogether 17,000 special envelopes and leaflets were distributed, and about 80 per cent. of these were returned with contributions, averaging from 1½d. per envelope in the poorest quarters to 1s. 9d. per envelope in the best streets. The total yield was £200 as against £133 in the previous year. This result is very satisfactory, but it remains to be seen whether when its novelty wears off it will still be regarded as distinct from those specially written appeals so often disregarded by householders.

### IMPORTANT EXTENSION TO THE WOMEN'S HOSPITAL, BIRMINGHAM.

When two years ago the Birmingham and Midland Women's Hospital built a small ward with eight beds for septic cases they perhaps hardly foresaw how much this experiment would be extended. Even then, however, its success was promptly shown by the number of applications from local practitioners for beds for puerperal fever patients. And thus the Women's Hospital entered the existing breach. The need for such action was increased as soon as the City Council decided to make puerperal fever a notifiable disease, and the Council arranged to have the disposal of two beds in the Women's Hospital in return for an annual grant of £320. Months have gone by and the demand on this department has grown so steadily that a scheme has now been approved for an additional 25 beds, 14 of which will be reserved for puerperal patients from Birmingham. In return the City Council will pay the institution £1,008, at the rate, that is, of £72 per bed per annum. The remaining beds will be for septic cases generally. The cost of the extension is expected to be £4,000, for which a public appeal will be made, and the architect is Mr. F. W. Martin, of Birmingham.

### THIS WEEK'S DRUG MARKET.

At the time of writing there has been no further development in the Quinine market, but a good business has been done in anticipation of an advance in price. There has been a good demand for eucalyptus oil at dearer rates. Ergot of rye is materially higher in value in consequence of the failure of the Spanish crop. Quicksilver has been reduced in price. Oil of cloves is rather dearer. Cream of tartar and tartaric acid are tending higher in value. The high price of balsam tolu is maintained. Ipecacuanha is dearer, as also is menthol. Almond oil has been slightly reduced in price. Rhubarb is tending dearer, and the stock is small. High prices are asked for cardamoms.

### TO OUR READERS.

Contributions are specially invited from any of our readers to these columns. They should deal with topical subjects and news. They must be authenticated for the information of the Editor only. The minimum payment if published is 5s. There is no hard-and-fast rule as to space, but notes of about twenty lines in length are preferred.

